# Gallbladder adenocarcinoma with sarcoid-like reaction in regional lymph nodes: report of a case

**DOI:** 10.1186/1471-2407-14-946

**Published:** 2014-12-13

**Authors:** Yota Kawasaki, Kosei Maemura, Hiroshi Kurahara, Yuko Mataki, Satoshi Iino, Masahiko Sakoda, Shinichi Ueno, Hiroyuki Shinchi, Sonshin Takao, Shoji Natsugoe

**Affiliations:** Department of Digestive Surgery, Breast and Thyroid Surgery, Graduate School of Medical Sciences, Kagoshima University, Sakuragaoka 8-35-1, Kagoshima, 890-8520 Japan

**Keywords:** Sarcoid-like reaction, Gallbladder cancer, Multiple swollen lymph nodes

## Abstract

**Background:**

Sarcoid-like reaction is often seen in various types of carcinoma, not only in the primary tumor, but also in regional lymph nodes, and can occur at any time, not only at the time of diagnosis, but also after treatment. However, few cases of hepatopancreatobiliary carcinoma, and no cases of gallbladder cancer with sarcoid-like reaction involving the lymph nodes have been described. This report is the first report of a sarcoid-like reaction involving the lymph nodes in a case of gallbladder cancer.

**Case presentation:**

We encountered a rare case of gall bladder cancer with sarcoid-like reaction in the lymph nodes. Since regional lymph node swelling that was difficult to differentiate from metastasis was found preoperatively, swollen nodes were examined histologically using frozen sections. Based on this histology, the swollen nodes were diagnosed as showing sarcoid reaction and therefore extended lymphadenectomy was avoided. The patient did not receive any adjuvant chemotherapy and has shown no recurrence of disease as of 4 years after surgery.

**Conclusion:**

Distinguishing between metastasis and sarcoid-like reaction in lymph nodes by preoperative imaging is still difficult. The present case shows that it is important to histologically examine swollen nodes by biopsy or by sampling before deciding on the treatment strategy for gall bladder cancer with swollen lymph nodes.

## Background

Sarcoid-like reaction is known to occur in patients with malignant disease and can occur at any time, not only at the time of diagnosis, but also after treatment [1]. Sarcoid-like reaction can be found in the stroma of the primary tumor itself, in lymph nodes draining the area of the primary tumor, and even in organs such as the liver, spleen, stomach and lungs [1–3].

Pathologically, sarcoid-like reaction includes non-caseating epithelioid cell granuloma that is comprised of a focal accumulation of epithelioid cells and multinucleated giant cells, and patients with sarcoid-like reaction show no clinical symptoms of systemic sarcoidosis. The non-caseating epithelioid granuloma is a result of antigen-specific cell-mediated immunity caused by macrophages and T-lymphocytes [4]. Although the pathogenesis of sarcoid-like reaction has been reported, no consensus has been reached on a complete definition. Sarcoid-like reaction is hypothesized to represent an antineoplastic immune phenomenon caused by antigenic factors derived from the tumor, with the high reactivity of the host immune system against such antigenic factors leading to the development of non-caseating epithelioid granuloma [2, 5].

Although many authors have reported sarcoid-like reactions in cancer patients, this finding appears to be very rare in patients with hepatopancreatobiliary cancer [6]. We report herein a rare case of gall bladder cancer with sarcoid reaction in the lymph nodes and no systemic sarcoid phenomenon.

## Case presentation

A 73-year-old Japanese woman was referred to our institution after a gall bladder mass was detected on screening ultrasonography (US).

**Figure 1:**
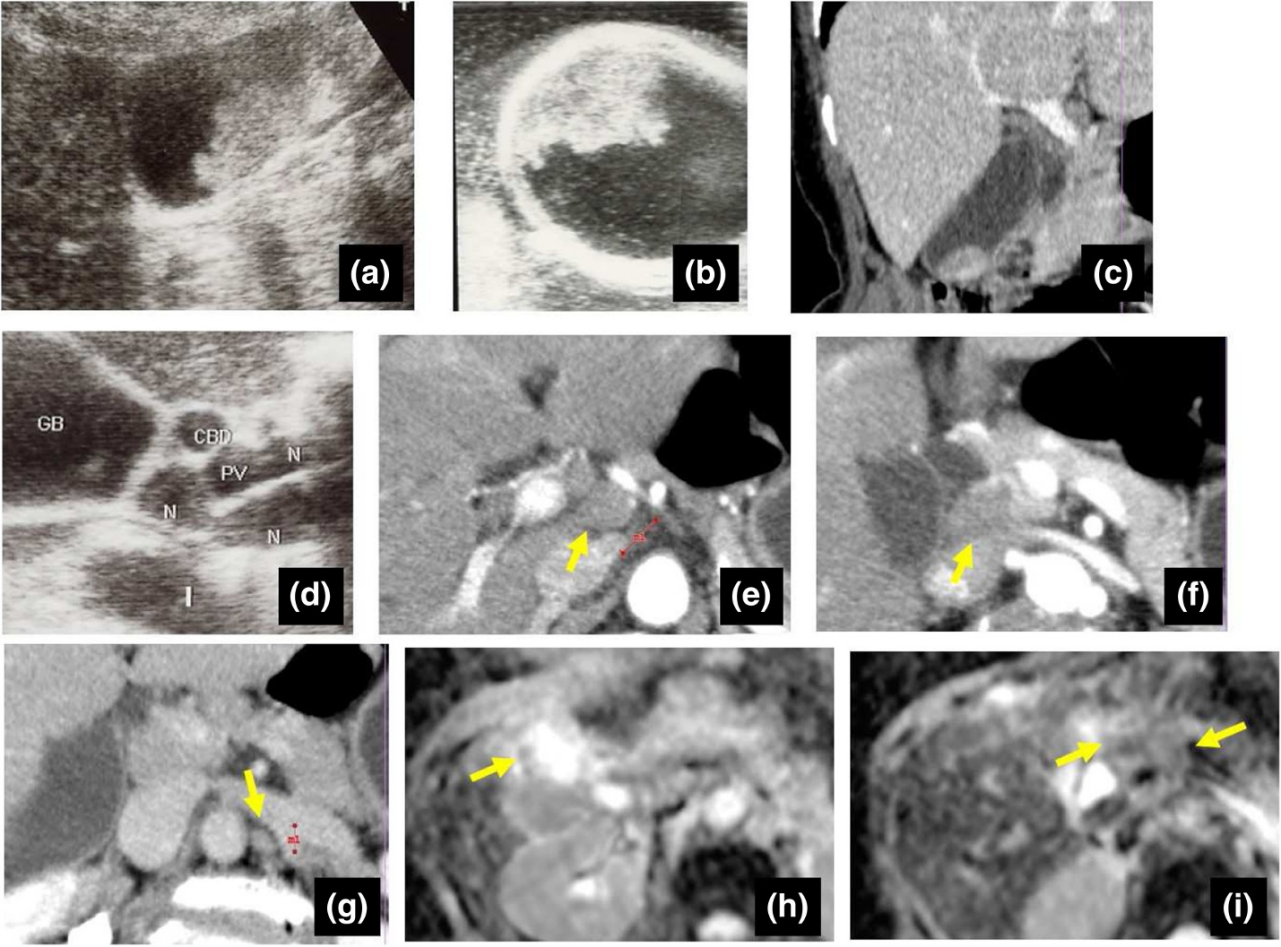
**Pre- and intraoperative images. a-c)** Ultrasonography (US) shows a highly echoic mass in the gallbladder. The size was φ30 mm. The widespread lesion showed irregular, gross progression, corresponding to gallbladder cancer. Because the outer layer of the gallbladder was clearly preserved (especially on intraoperative US), indicating Type B, tumor depth was estimated as extending to the muscularis propria. Computed tomography (CT) also shows a mass in the gallbladder, with no invasion to other organs. **d)** US shows multiple swollen lymph nodes in the hepatoduodenal ligament. **e-g)** CT shows swelling of not only lymph nodes at the hepatoduodenal ligament, but also para-aortic lymph nodes. **h,i)** Diffusion-weighted imaging (DWI) shows signal hyperintensity for the gallbladder mass, but signal hypointensity in all detected swollen lymph nodes.

The patient had no complaints, and both physical examination and laboratory findings (including carcinoembryonic antigen, carbohydrate antigen 19–9, and soluble interleukin-2 receptor) were unremarkable. When we performed US, a mass measuring 23 × 22 mm was identified at the fundus of the gall bladder. This broad-based, sessile mass showed preservation of the outer hyperechoic layer, suggesting gall bladder cancer. According to a previous report, these findings corresponded with a Type B image [7]. Tumor invasion seemed limited to the muscularis propria (cT1b) [7]. Computed tomography (CT) revealed several lymph nodes that were swollen to 7–15 mm, located at both the hepatoduodenal ligament and in the para-aortic area. Diffusion-weighted imaging (DWI) showed a signal-hyperintense gallbladder mass, but signal hypointensity for all detected swollen lymph nodes (Figure 1). Based on such findings, the swollen nodes were diagnosed as metastases. As a preoperative summary, we regarded this tumor as clinically stage IIB (cT1b, cN1, cM0) based on the 7^th^ edition of the Union for International Cancer Control (UICC).

**Figure 2:**
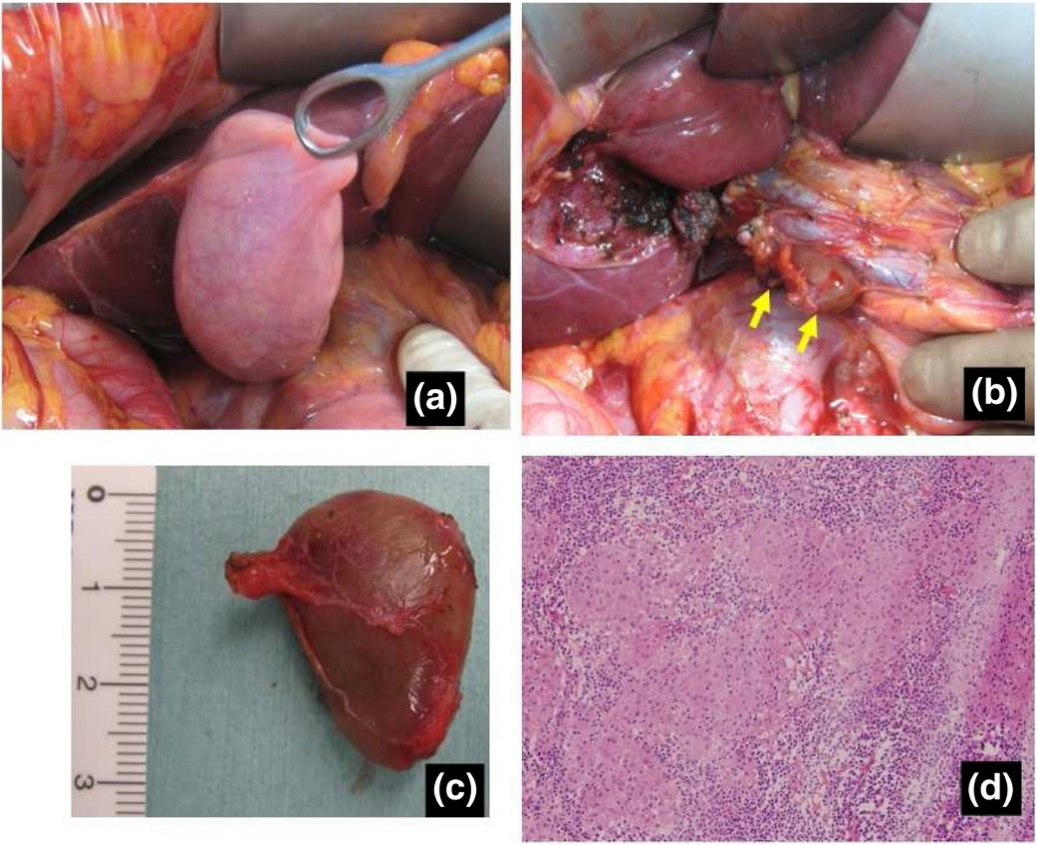
**Intraoperative findings. a)** Macroscopically, no invasion to the serosa is evident. No shrinkage derived from invasion to the subserosa is seen. **b,c)** Swollen lymph nodes are easily dissected out, and the margins are clear. **d)** Frozen-section examination of swollen nodes shows only non-caseating epithelioid cell granuloma.

**Figure 3:**
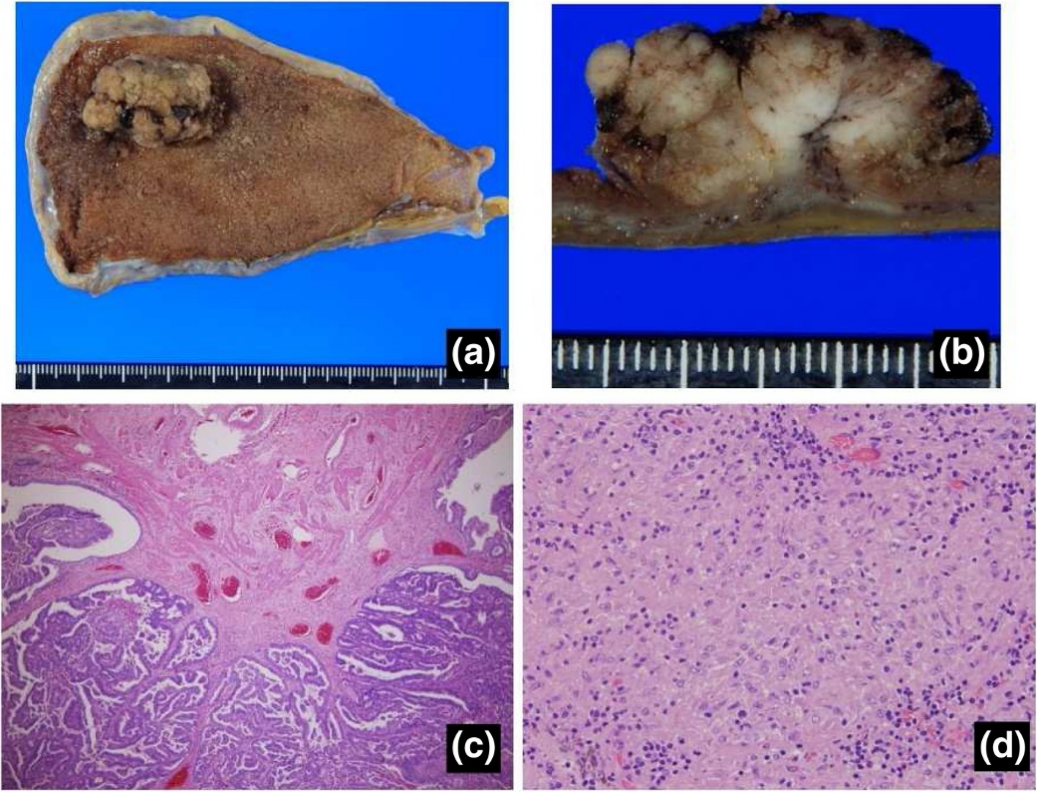
**Macro- and microscopic findings. a)** Macroscopic appearance of the gall bladder mass located in the fundus. **b)** Tumor infiltration to the muscularis layer is evident macroscopically. **c)** Tumor infiltration to the muscularis propria is evident microscopically. **d)** Final pathological examination of swollen lymph nodes shows only non-caseating epithelioid cell granuloma, with no evidence of metastasis in any dissected lymph node.

**Figure 4:**
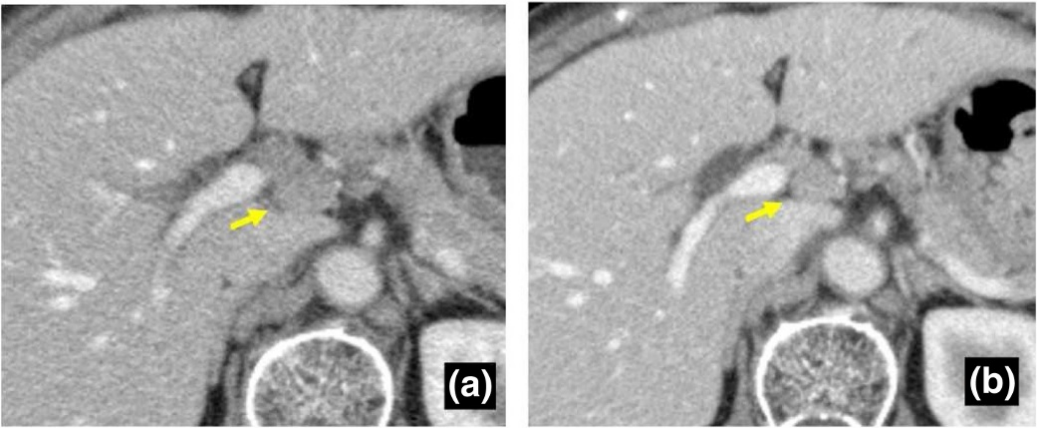
**Course of remaining swollen lymph nodes. a)** CT 5 months after surgery shows a remaining swollen lymph node (No. 12). The size is 15 × 11 mm. **b)** CT 46 months after surgery also shows the remaining swollen No. 12 lymph node. The size (11 × 8 mm) appears to be reduced compared to the initial examination.

Based on the above evidence of gallbladder cancer, we planned to perform radical cholecystectomy involving removal of the gallbladder and extrahepatic biliary tract with en-bloc subsegmental resection of the adjacent hepatic parenchyma of segments 4B and 5, and regional lymphadenectomy involving complete removal of the hepatoduodenal ligament lymph nodes, common hepatic artery nodes and retropancreatic nodes [8]. During surgery, we detected swollen lymph nodes. Four of these lymph nodes were dissected and frozen sections were histologically examined. Unlike typical metastatic lymph nodes, these nodes showed a smooth, round shape with clear margins, and were easy to dissect (Figure 2). Histological frozen-section examination revealed that none of the lymph nodes showed tumor involvement but instead they showed non-caseating epithelioid cell granuloma (Figure 2). We therefore only performed simple cholecystectomy, and some of the swollen nodes in the hepatoduodenal ligament were not dissected. Histological examination of the resected gall bladder showed papillary carcinoma with invasion limited to the muscularis propria. None of the dissected lymph nodes showed tumor involvement and all were diagnosed as showing non-caseating epithelioid cell granuloma. Accordingly, the patient was diagnosed with papillary adenocarcinoma of the gall bladder, stage IA (pT1b, pN0, pM0) (Figure 3). Because of the early stage of gall bladder cancer, adjuvant chemotherapy was not performed. As of 4 years postoperatively, the patient has shown no disease recurrence. During follow-up examination, the size of swollen nodes remaining in the hepatoduodenal ligament has gradually decreased compared to the initial size at the time of surgery (Figure 4).

### Discussion

Sarcoid-like reaction related to a tumor was first reported in the early 19^th^ Century [5]. Since then, such reactions have been reported with various carcinomas, such as those of the stomach, lungs, uterus and breast [3, 9, 10]. Brincker et al. [5] reported that sarcoid-like reactions may occur in 4.4% of cancer patients, 13.8% of patients with Hodgkin’s disease, and 7.3% of patients with non-Hodgkin lymphoma. In cases of carcinoma, sarcoid-like reaction is found in regional lymph nodes, particularly in non-metastatic lymph nodes. Few cases of hepatopancreatobiliary carcinoma with sarcoid-like reaction involving the lymph nodes have been described. Fong et al. [6] summarized 15 cases of hepatopancreatobiliary tumor with sarcoid-like reaction in 2012. However, only 19 cases in total have been reported to date (Table 1), and there have been no reports regarding sarcoid-like reaction in gallbladder cancer.

**Table 1:** **Reports of sarcoid-like reactions in patients with hepatopancreatobiliary malignancy**

Author	Year published	Journal	Age	Sex	Primary disease	Location of sarcoid reaction
Herxheimer G [11]	1917	Z Tuberk	N/A	N/A	Cholangiocarcinoma	Liver
Gherardi G [12]	1950	Arch Pathol	61	F	Cholangiocarcinoma	Peri-hepatic LN
Nadel EM [13]	1950	Am J Clin Pathol	64	M	Ampullary adenocarcinoma	Peri-pancreatic LN
Ten Seldam RE [14]	1956	Med J Australia	N/A	N/A	Cholangiocarcinoma	Lesser Omental LN
Muto Y [15]	1982	Jpn J Gastroenterol Surg	62	F	Cholangiocarcinoma	Hilar and pericystic LN
Schmidt D [16]	1985	Virchow Arch A Othhol Anat Hitopathol	N/A	N/A	Hepatoblastoma	Liver
Schmidt D [16]	1985	Virchow Arch A Othhol Anat Hitopathol	N/A	N/A	HCC	Liver
Van Steenbergen W [17]	1987	J Clin Gastroenterol	30	M	Cholangiocarcinoma	Liver and Hilar LN
Klein M [18]	1994	Chest	61	M	Cholangiocarcinoma	Hilar and paratracheal LN
Nakao A [19]	1996	Biliary Tract Pancreas	69	F	Cholangiocarcinoma	Peri-ductal LN
Shito M [20]	1997	Jpn J Surg	69	F	Cholangiocarcinoma	Peri-ductal LN
Onitsuka A [21]	2003	J Hepatobiliary Pancreat Surg	74	F	Cholangiocarcinoma	Peri-ductal LN
Mao JT [22]	2000	Am J Med	71	M	PDA	Lung
Kurata A [23]	2005	Hum Pathol	44	M	PDA	Peri-pancreatic LN
Kurata A [23]	2005	Hum Pathol	60	M	PDA	Peri-pancreatic LN
Chowdhury FU [2]	2009	Clin Radiology	62	M	PDA	Sub-pleural LN
Fong ZV [6]	2012	J Gastrointest Surg	59	F	Cholangiocarcinoma	Spleen
Fong ZV [6]	2012	J Gastrointest Surg	68	F	IPMN	liver and bone
Mastroroberto M [24]	2012	Journal of the pancreas	52	M	NET, pancreas	Hilar and paratracheal LN

PDA: pancreatic adenocarcinoma, IPMN: intraductal papillary mucinous neoplasm, NET: neuroendcrine tumor F: female, M: male, LN: lymph node.

Gallbladder cancer is the most common malignant neoplasm of biliary tract cancer, and unfortunately the prognosis is well known to be very poor [25]. In many cases, gall bladder cancer is already at an advanced stage by the time of detection and diagnosis [8]. Lymph node metastasis is an important prognostic factor for this tumor, as it is for other malignant tumors. Preoperative diagnosis of the presence or absence of nodal metastasis is thus important when planning a surgical strategy. In the case of node positive gallbladder cancer (except for positive para-aortic lymph nodes), although there is certainly some controversy, it is considered that “aggressive” radical surgery such as combined resection of the liver and the common bile duct together with extended lymphadenectomy might contribute to better prognosis [26, 27]. In the present case, we preoperatively diagnosed multiple nodal swellings observed by imaging as positive evidence of metastasis. Distinct radiographic patterns that can distinguish sarcoid-like reaction, sarcoidosis and carcinoma have not yet been identified [1]. If the differential diagnosis of lymph node swelling is difficult, intraoperative histological examination of frozen sections is essential. In this case, we were able to avoid extended lymphadenectomy, because the histological results showed a sarcoid-like reaction. Some authors have reported that preoperative biopsy through endoscopic ultrasound-guided-fine needle aspiration (EUS-FNA) may represent a useful method for analysis of such nodes [1, 28, 29].

The medical records of this patient included no symptoms or signs related to systemic sarcoidosis, such as erythema nodosum, uveitis, acute polyarthritis, respiratory illness, fatigue, malaise, weight loss or fever [30]. Moreover, no serum abnormalities such as hypercalcemia or elevated levels of angiotensin-converting enzyme were seen. Accordingly, we speculate that the development of non-caseating epithelioid cell granuloma in this patient was not caused by systemic sarcoidosis, but rather by a sarcoid-like reaction related to gallbladder cancer. Sarcoid-like reactions are comprised of different inflammatory cells, mature dendritic cells and T lymphocytes in non-caseating epithelioid cell granulomas [23]. Infiltration of tumors by dendritic cells reflects a host immune defense mechanism [31]. It is well known that the presence of dendritic cells and T lymphocytes in tumors is associated with better prognosis, reduced tumor recurrence and fewer metastases [32–36]. Although no consensus has been reached, these evidences seem to suggest that patients with a malignant tumor that shows a sarcoid-like reaction may have a better prognosis [37]. Indeed, our patient has shown long-term survival and no recurrence after surgery.

Few reports have focused on the course of swollen nodes after resection of a primary cancer. Interestingly, in this case, the residual swollen nodes gradually decreased in size after resection of the gallbladder cancer. This phenomenon clinically supports the view that sarcoid-like reaction results from high reactivity of the host immune system against antigenic factors from the primary cancer.

## Conclusion

We encountered a case of sarcoid-like reaction related to gallbladder cancer. Distinguishing between metastasis and sarcoid-like reaction in lymph nodes by preoperative imaging is still difficult. The present case shows that it is important to histologically examine swollen nodes by biopsy or sampling before deciding on the treatment strategy for gall bladder cancer with swollen lymph nodes.

### Consent

Written informed consent was obtained from the patient for publication of this Case report and any accompanying images. A copy of this written consent is available for review by the Editor-in-Chief of this journal. This study was approved by the institutional ethics review board of Kagoshima University Hospital (reference number 25–39).

## Abbreviations

US: Ultrasonography
CT: Computed tomography CT
DWI: Diffusion-weighted imaging DWI
UICC: Union for International Cancer Control
EUS-FNA: Endoscopic ultrasound-guided-fine needle aspiration.
